# A Self-Assembly Portable Mobile Mapping System for Archeological Reconstruction Based on VSLAM-Photogrammetric Algorithm

**DOI:** 10.3390/s19183952

**Published:** 2019-09-12

**Authors:** Pedro Ortiz-Coder, Alonso Sánchez-Ríos

**Affiliations:** Department of Graphic Expression, University Centre of Mérida, University of Extremadura, 06800 Mérida, Spain

**Keywords:** self-assembly device, 3D point clouds, accuracy analysis, VSLAM-photogrammetric algorithm, portable mobile mapping system, low-cost device, BIM

## Abstract

Three Dimensional (3D) models are widely used in clinical applications, geosciences, cultural heritage preservation, and engineering; this, together with new emerging needs such as building information modeling (BIM) develop new data capture techniques and devices with a low cost and reduced learning curve that allow for non-specialized users to employ it. This paper presents a simple, self-assembly device for 3D point clouds data capture with an estimated base price under €2500; furthermore, a workflow for the calculations is described that includes a Visual SLAM-photogrammetric threaded algorithm that has been implemented in C++. Another purpose of this work is to validate the proposed system in BIM working environments. To achieve it, in outdoor tests, several 3D point clouds were obtained and the coordinates of 40 points were obtained by means of this device, with data capture distances ranging between 5 to 20 m. Subsequently, those were compared to the coordinates of the same targets measured by a total station. The Euclidean average distance errors and root mean square errors (RMSEs) ranging between 12–46 mm and 8–33 mm respectively, depending on the data capture distance (5–20 m). Furthermore, the proposed system was compared with a commonly used photogrammetric methodology based on Agisoft Metashape software. The results obtained demonstrate that the proposed system satisfies (in each case) the tolerances of ‘level 1’ (51 mm) and ‘level 2’ (13 mm) for point cloud acquisition in urban design and historic documentation, according to the BIM Guide for 3D Imaging (U.S. General Services).

## 1. Introduction

The tridimensional modeling of an object starts with its original design or with the process of acquiring the data necessary for its geometric reconstruction. In both cases, the result is a 3D virtual model that can be visualized and analyzed interactively on a computer [1,2]. In many cases, the process continues with the materialization of the model in the form of a prototype, which serves as a sample of what will be the final product, allowing us to check if its design is correct, thus changing the traditional manufacturing or construction industry [3,4,5].

The applications of 3D models (virtual or prototype) are numerous and widely used; they are usually used in the scope of clinical applications [6,7], geosciences [8,9,10,11,12,13], cultural heritage preservation [14,15] and engineering [16].

In this context, to address this wide variety of application areas, both data capture techniques and devices, as well as the specific software for data processing and management tend to be simplified. This is done in order to be accessible to the greatest number of users, even with limited knowledge in 3D measurement technologies.

In this sense, the classical methods of photogrammetry are combined with new techniques and procedures which are usually adopted for other areas [17], such as visual odometry (VO), the simultaneous localization and mapping (SLAM) and the visual slam (VSLAM) techniques. These are normally used to solve localization and mapping problems in the areas of robotics and autonomous systems [18,19,20,21], but also the combination of photogrammetry techniques with methodologies based on instruments like terrestrial or aerial laser scanners have obtained successful results [22,23]. 

These combined methods provide support and analytical robustness for the development of low/middle-cost capture systems, usually based on tablets or mobile devices that incorporate inertial sensors, absolute positioning and low cost cameras which can achieve medium 3D positional accuracy scanning, in compliance with technical requirements of a wide range of applications at a low cost and reduced learning curve [24,25,26,27,28]. As a result, handheld mobile mapping systems have appeared in recent years, using different technologies to perform 3D reconstructions that use fully automated processes [27,28]. Among which we can find systems based exclusively on images, requiring a fully automated process, taking into account the usual technical constraints in photogrammetry, and the free user movements in data capture [17]. In this field, different lines of research have been developed, depending on whether the final result is obtained in real time [29,30] or not. In the first case, the reduction in the time needed for data processing is the most important factor in the approach to research objectives (even at the expense of a metric accuracy reduction); in the second, however, metric accuracy is the most important factor, although the temporal cost is higher [17,31,32].

There are many commercial mobile mapping systems for urban, architectural or archaeological applications with high accuracy results [33]. Those systems are based on the integration on different sensors such as (Inertial Measurement Unit) IMU, line scanners [28], cameras, Global Navigation Satellite System (GNSS) [34], odometers and other sensors. The price and complexity of those systems are normally high [35].

The classical applications require a known level of data accuracy and quality, however, the emerging needs of Industry 4.0, building information modeling (BIM) or digital transformation, next to the appearance of new devices and information processing techniques pose new challenges and research opportunities in this field. Each capture method has its advantages and drawbacks, offering a particular level of quality in its results; in this sense, numerous investigations have linked these parameters, allowing people to choose the most cost-effective approach [35]. This can be achieved by way of evaluating the use of the laser scanner and the vision-based reconstruction among different solutions for progress monitoring inspection in construction and concluding (among other characteristics) that both of them are appropriate for spatial data capture. This could [36] include among 3D sensing technologies, photo/video-grammetry, laser scanning and the range of images that make a detailed assessment of the content (low, medium or high) into BIM working environments. It may also include [37] comparing photo/video-grammetry capture techniques with laser scanning, considering aspects such as accuracy, quality, time efficiency and cost needed for collecting data on site. The combination of data capture methods has also been traditionally analyzed; thus, [38] presently, there is a combined laser scanning/photogrammetry approach to optimize data collection, cutting around 75% of the time required to scan the construction site.

A common aspect, taken into account in most of the research, is the point cloud accuracy evaluation, that has been addressed in three different ways [39]: (a) By defining levels of quality parameters defined in national standards and guidelines that come from countries like the United States, Canada, the United Kingdom and Scandinavian countries that lead BIM implementation in the world [40], such as the U.S. General Services Administration (GSA) BIM Guide for 3D Imaging [41] that sets the quality requirements of point clouds in terms of their level of accuracy (LOA) and level of detail (LOD); (b) by evaluating quality parameters of a point cloud, in terms of accuracy and completeness [37] or (c) following three quality criteria: Reference system accuracy, positional accuracy and completeness [42].

Furthermore, in the specific environment of 3D indoor models, [43] we propose a method that provides suitable criteria for the quantitative evaluation of geometric quality in terms of completeness, correctness, and accuracy by defining parameters to optimize a scanning plan in order to minimize data collection time while ensuring that the desired level of quality is satisfied, in some cases, with the implementation of an analytical sensor model that uses a “divide and conquer” strategy based on segmentation of the scene [44], or one that captures the relationships between parameters like data collection parameters and data quality metrics [45]. In other cases, the influence of scan geometry is considered in order to optimize measurement setups [46], or are compared to different known methods for obtaining accurate 3D modeling applications, like in the work of [47], in the context of cultural heritage documentation.

This paper extends on past surveys of classical photogrammetry solutions, adopting an extended solution approach for outdoor environments based on the use of a simple and hand-held self-assembly device for data capture, based on images, that consist on two cameras: One, which data will be used to calculate in real time, the path followed by the device using a VSALM algorithm, while with other one; a high-resolution video recorded and used to achieve the scene reconstruction using photogrammetric techniques. Finally, after following simple data collection and fully automated processing, a 3D point cloud with associated color is obtained.

To determine the effectiveness of the proposed system, we evaluate it in one study site performed outdoors in the facades of the Roman Aqueduct of Miracles, in terms of the requirements laid down in the GSA BIM Guide for 3D Imaging. In this experiment, we obtain 3D point clouds from different data capture conditions, that vary according to the distance from the device and the monument; the measurements acquired by a total station serve to compare the coordinates of fixed points in both systems, and therefore, determining the LOA of each point cloud. The results obtained, with root mean square errors (RMSEs) between eight and 33 mm, stress the feasibility of the proposed system for urban design and historic documentation projects, in the context of allowable dimensional deviations in BIM and CAD deliverables.

This paper is divided into four sections. Following the Introduction, the portable mobile mapping system is described, including the proposed algorithm schema for the computations; therefore, a case study in which the system is applied is described in Section 2. The results are presented in Section 3 and finally, the conclusions are presented in Section 4.

## 2. Materials and Methods 

**Figure 1:**
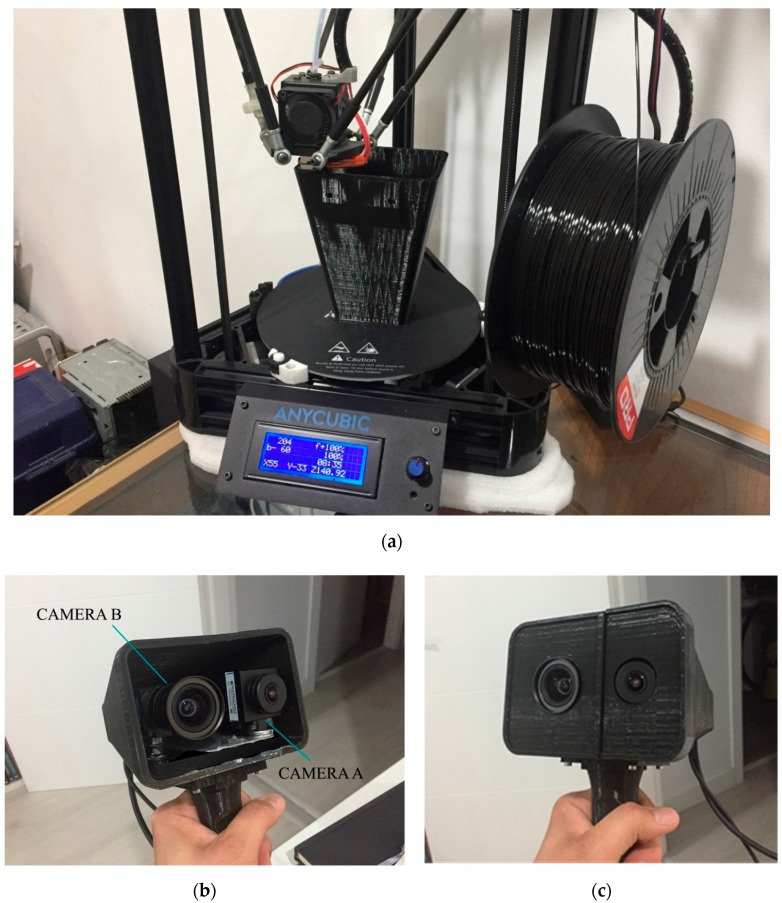
(**a**) 3D printing process of the prototype case; (**b**) cameras A and B with their placement inside the case; and (**c**) the final portable mobile mapping system prototype.

This study was conducted with a simple and self-assembly prototype specifically built for data capture (Figure 1), that consists of two cameras from the Imaging Source Europe GmbH company (Bremen, Germany): Camera A (model DFK 42AUC03) and camera B (model DFK 33UX264) were fixed to a platform with the condition of its optical axes being parallel; each camera incorporated a lens; for camera A, the model was TIS-TBL 2.1 C, from the Imaging Source Europe GmbH company and for camera B, the model was the Fujinon HF6XA–5M, from FUJIFILM Corporation (Tokyo, Japan). The technical characteristics of cameras and lenses appear in Table 1 and Table 2, respectively. Both cameras were connected to a laptop (with an Intel core i7 7700 HQ CPU processor and RAM of 16 Gb, running under Windows 10 Home), via USB 2.0 (camera A) and 3.0 (camera B). This beta version of the prototype had an estimated base price under €2500.

The calibration process of the cameras was carried out with a checkerboard target (60 cm × 60 cm) using a complete single camera calibration method [48], that provided the main internal calibration parameters: The focal length, radial and tangential distortions, optical center coordinates and camera axe skews. In addition, to know the parameters that related to the position of one camera compared to the other, we designed the following, practical test: To use as ground control points we placed 15 targets on two perpendicular walls and measured the coordinates of each target with a TOPCON Robotic total station, with an accuracy of 1” measuring angles (ISO 17123-3:2001) and 1.5 mm + 2 ppm measuring distances (ISO 17123-4:2001). After running the observations with the prototype, we used a seven-parameter transformation, using the 15 targets, to determine the relative position of one camera in relation to the other [17].

Camera A and camera B had different configuration parameters which defined image properties such as brightness, gain or exposure between others. In order to automate the capture procedure, automatic parameters options had been chosen. In such a way, the data collection was automatic and the user didn’t need to follow specials rules since the system accepted convergent or divergent turns of the camera, stops or changes in speed. The algorithm processed all this data properly using the proposed methodology.

**Figure 2:**
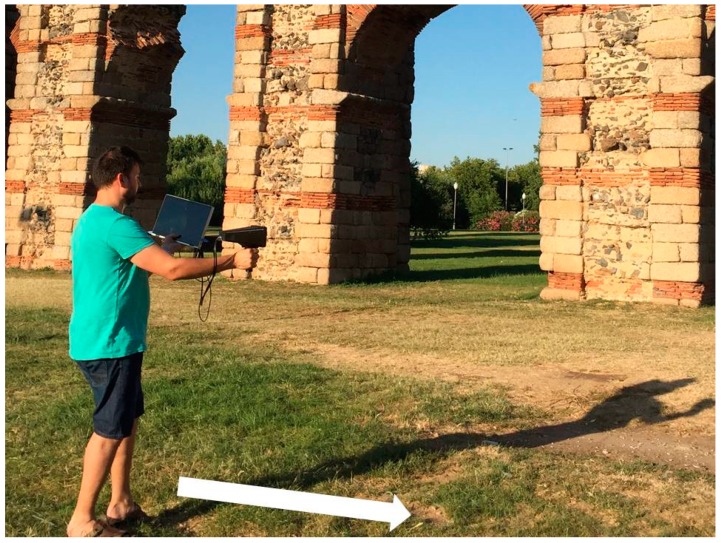
In-field data capture. The white arrow indicates the user direction movement, parallel to the monument façade, followed during this test.

During the capture (Figure 2), the user needed to see the VSLAM tracking in the screen of the computer in real time. In this way, the user was sure he didn´t make a fast movement or if an item appeared that interrupted camera visualization and, therefore, the tracking could not continue. In this case, the user must return again to a known place and continue the tracking from this point.

### Workflow of the Proposed Algorithm for the Computation

The application of the VSLAM technique on a low weight device, normally with limited calculation capabilities, needed the implementation of a low computational cost VSLAM algorithm to achieve effective results. The technical literature provided a framework that consisted of the following basic modules: The initialization module; to define a global coordinate system, and the tracking and mapping modules; to continuously estimate camera poses. In addition, two additional modules were used for a more reliable and accurate result: The re-localization module, that has to be used when, due to a fast device motion or some disruptions in data capture, the camera pose must be computed again and the global map optimization, which is performed to estimate and remove accumulative errors in the map, produced during camera movements.

**Figure 3:**
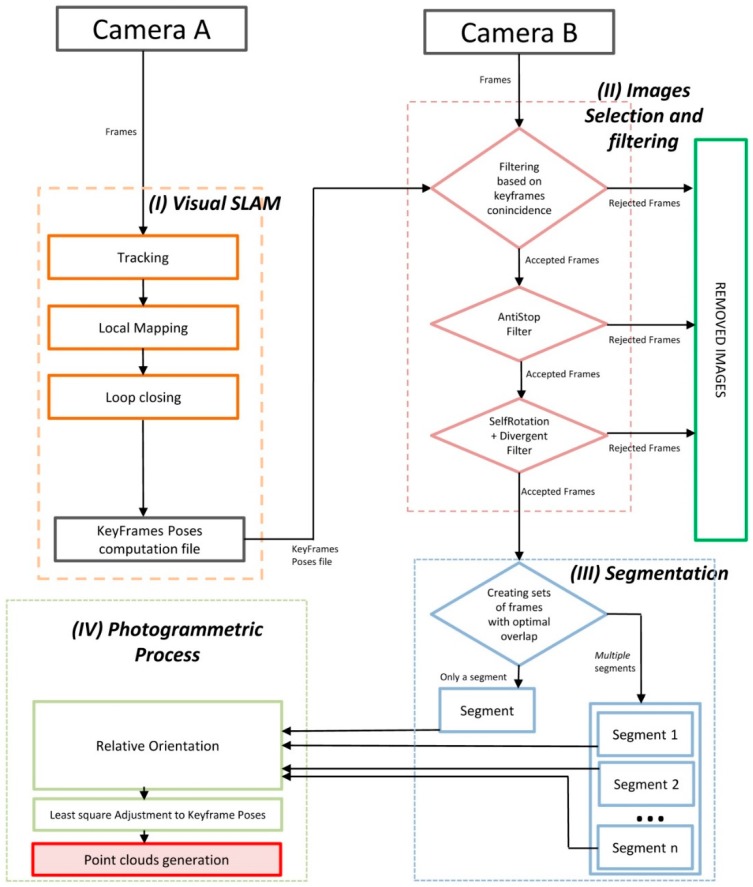
General workflow of the algorithm implemented in C++ for the computations.

The characteristics of the VSLAM-photogrammetric algorithm, including identified strong and weak points, depend on the methodology used for each module which sets its advantages and limitations. In our case, we proposed the following sequential workflow (Figure 3) divided into four threaded processes, which have been implemented in C++.

Basically, the four processes consisted in the following: (I) A VSLAM algorithm to estimate both motion and structure, that is applied in frames obtained from camera A, (II) an image selection and filtering process of frames obtained with camera B, (III) the application of an image segmentation algorithm and finally, (IV) a classical photogrammetric process applied to obtain the 3D point cloud. Each process is explained in more detail below.

The first process (I) started with the simultaneous acquisition of videos with cameras A and B, with speeds of 25 FPS and 4 FPS, respectively. With the frames from camera A, used as an ORB descriptor [49] for object recognition, detection and matching was used. This descriptor built on the FAST key-point detector and the BRIEF descriptor, with good performance and low cost, and therefore, was appropriate for our case. An ORB-SLAM algorithm was then applied to estimate camera positioning and trajectory calculation [50]; this was an accurate monocular SLAM system that worked in real time and can be applied in indoor/outdoor scenarios, and has modules to loop closure detection, re-localization (to recover from situations where the system becomes lost) and to a totally automatic initialization, taking into account the calibration parameters of the camera. From these remarks, our process was carried out in three steps as follows [50]. The first step was the tracking, which calculated the positioning of the camera for each frame and selected keyframes and decided which frames were added to the list; the second one was local mapping, which performed keyframes optimization, incorporating those that were being taken and removing the redundant keyframes. With these data, through a local bundle adjustment, in addition to increasing the quality of the final map, it was possible to reduce the computational complexity of the processes that were just running, and equally for the subsequent steps. The third one was loop closing, which looked for redundant areas where the camera had already passed before, which could be found in each new keyframe; the transformation of similarity on the accumulated drift in the loop was calculated, the two ends of the loop were aligned [50], the duplicate points were merged and the trajectory was recalculated and optimized to achieve overall consistency. The result of this process was a text file with UNIX time parameters and camera poses of the selected keyframes.

The above information together with the frames recorded by camera B, was used to start the second process (II), in which a selection and filtering of the images obtained by camera B was carried out, which consisted in the direct deletion of images whose baseline was very small, and therefore, which made it difficult to compute an optimum relative orientation [17,51,52]. The filtering process was performed in three consecutive steps: The first, a filtering based on keyframes coincidence, which consisted of incorporating a *β* number of frames (in our case *β* = 2) from camera B between each two consecutive frames from camera A and, at the same time, the remaining frames were removed. To run this filter, it was necessary that the cameras were synchronized by UNIX time. The second process, applied the so-called AntiStop filter, which removed those frames obtained in the event that the camera had been in a static position, or with a very small movement, recorded images of the same zone, which we described as redundant and which should, therefore, be eliminated. To determine the redundant frames, it was assumed that cameras A and B were synchronized and that we knew the coordinates of the projection center of each frame, computed in (I). We continued with the calculation of the distances between the projection centers of every two consecutive keyframes *i* and *j* (*D_ij_*) as well as the mean value of all the distances between consecutive frames (*D_m_*) and the definition of the minimum distance (*D_min_*) from which the device was either stopped or was in motion, by the expression:

*D_min_* = *D_m_* * *p*,

where *p* is a parameter that depends on the data capture conditions (in our case, after performing several tests, we defined a value of *p* = 0.7). Finally, the keyframes took by camera B in which the distance between the projection centers of each two consecutive keyframes was less than the minimum distance (*D_ij_* < *D*_min_) were removed.

**Figure 4:**
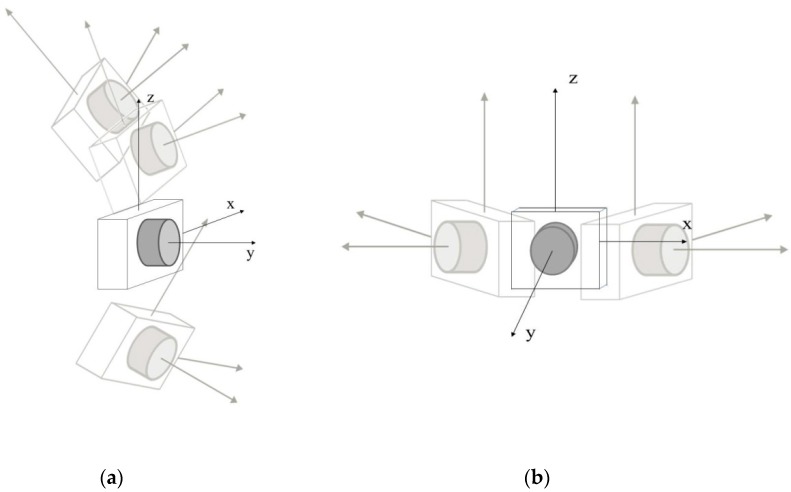
Divergent self-rotation in the (**a**) *X*-axis; and (**b**) *Z*-axis.

The third, called the divergent self-rotation filter, was able to remove those keyframes captured by camera B when they met two conditions: The rotation angles of the camera *ω_i_* (*X* axis) and *κ_i_* (*Z* axis) (Figure 4) increase or decrease their value permanently during data capture for of at least three consecutive frames at a value of ±9° (in our case), and besides, their projection centers were very close to each other; for the calculation of the same procedure is the same one used as the one used for the AntiStop filter, but with a different value of *p* (in our case, we considered a value of *p* = 0.9).

The next process (III) was segmentation, which aimed to obtain more significant and easy to analyze images in the subsequent photogrammetric process. It started searching for the homologous points belonging to the keyframes resulting from the filtering process carried out in (II) [53,54,55], which was performed between an image, the earlier one and the later one. The resulting images were stored in a set, called a “segment”. The result of this process generated one or more independent segments among themselves, which had a number of homologous points and an appropriate distribution to be properly oriented (in our case, 200 points and 10% of these points were in each quadrant of the image; in addition, if the segment did not have at least three images, it was discarded and its images were removed).

The last process (IV) was called the photogrammetric process, which was structured in three steps: The first was to compute a relative image orientation [53] setting the first image as the origin of the relative reference system and used the homologous points of each segment and algorithms leading to direct solutions [17,51,53]; then, a bundle adjustment) was used on the oriented images to avoid divergences [56], obtaining the coordinates of the camera poses and computed tie points. The second step consisted of an adjustment of the camera poses in each segment to adapt them to the overall trajectory, computed in (I). This procedure was performed using minimum square techniques [57] in each segment, and a three-dimensional transformation [10] to correct the positions of camera B with respect to camera A.

In the third step, the scene was reconstructed using MICMAC software [54], in order to obtain dense cloud points with color. MICMAC is a free open-source photogrammetry software developed by the French National Mapping Agency (IGN) and the National School of Geographic Sciences (ENSG) [58]. This software generates a depth map from the main image and a series of secondary images to obtain parallax values. The calculation was carried out having taken into account that the scene could be described by a single function *Z* = *f*(*X*; *Y*) (with *X*; *Y*; *Z* using Euclidean coordinates) with several parameters of MICMAC to calculate the density correlation and obtain the cloud of dense points with color [54,55,59] which was the final result of the process.

## 3. Accuracy Assessment and Results

**Figure 5:**
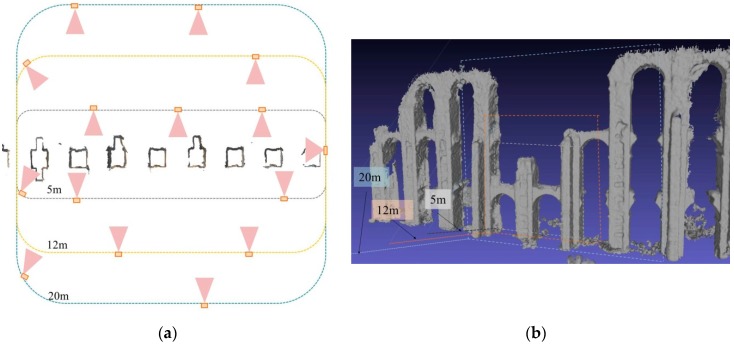
(**a**) Scheme with the data capture trajectories and (**b**) the areas covered by a frame, for 5, 12 and 20 m of distance prototype-monument. The figure that appears in (**b**), is a 3D model (mesh) generated by the software Meshlab [61] from the 20 m points cloud made only for visualization purposes.

This work determined the accuracy of a set of point clouds obtained with the prototype in order to validate the device for BIM work environments. Additionally, the results were compared with a usual photogrammetric procedure, using a reflex camera and photogrammetric software (Agisoft Metashape [60]), in order to compare the advantages and disadvantages of the proposed prototype in respect to this known methodology. For this, an experimental test was carried out in the Roman aqueduct of “The Miracles” in the city of Mérida (Spain). This monument, built in the first-century A.C, has a total dimension of 12 km in length between underground and aerial sections with arches. The test was carried out on an archery stretch which was 23 m high and 60 m wide, performing a set of three data capture scenarios at different observation distances (5, 12, and 20 m) from the prototype to the base of the monument (Figure 5).

In this test, the data collection was carried out in such a way that the movement of the user followed a perpendicular direction to the camera optical axe (Figure 2), avoiding divergent turns since this kind of movement was not necessary in this case. In this way, this prevented the algorithm from using the divergent self-rotation filter in an unnecessary situation.

**Figure 6:**
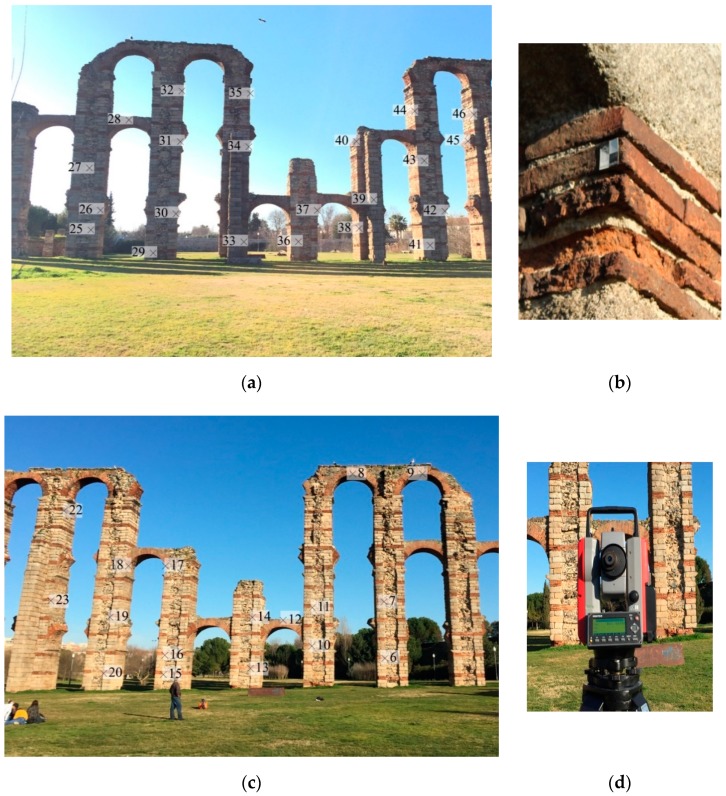
(**a**,**c**) Reference points spread on the two fronts of the monument. (**b**) Target model used in the test and (**d**) total station Pentax V-227N used to measure the network coordinates.

In order to evaluate the metric quality of the measures obtained with the prototype and the Agisoft Metashape photogrammetric procedure, a control network was performed to be used in the dimensional control study, following the procedures carried out by [62] and [63]. The network was used as reference points and consisted on a set of targets and natural targets whose three-dimensional coordinates in a local coordinate system were obtained by a second measuring instrument (more precise than the device we want to evaluate). In this case, a total station Pentax V-227N (Pentax Ricoh Imaging Company, Ltd, Tokyo, Japan) was used, with an accuracy of 7′ for angular measurements (ISO 17123-3:2001) and 3 mm ± 2 ppm for distance measurements (ISO 17123-3:2001) with which a total of 40 uniformly distributed targets have been measured (Figure 6).

Then, the method proposed by [62] was used, in which the accuracy of the 3D point cloud was quantified according to the Euclidean average distance error (*δ_avg_*) as:

(1) $$\delta_{avg}=\frac{1}{n}\overset{n}{\underset{i=1}{\sum }}|Ra_{i}-T-b_{i}|$$

where $a_{i}$ is the *i*th checkpoint measured by the prototype, $b_{i}$ is the corresponding reference point acquired by the total station, *R* and *T* are the rotation and translation parameters for 3D Helmert transformation.

And the quality of the 3D point cloud was also evaluated by the root mean square error (RMSE) as:

(2) $$RMSE=\sqrt{\frac{1}{n}\overset{n}{\underset{i=1}{\sum }}{\left(a_{i}^{t}-b_{i}\right)}^{2}}$$

where $a_{i}^{t}$ indicates the $a_{i}$ point after the 3D conformal transformation to bring the model coordinates in the same system of the reference points.

As mentioned previously in Section 1 of this paper, the point cloud accuracy evaluation can be done according to different criteria. In our case, we have used the GSA BIM Guide for 3D Imaging criteria, that defines four levels of detail (LOD) with dimensions of the smallest recognizable feature ranging between 13 mm × 13 mm to 152 mm × 152 mm; and also defines the level of accuracy (LOA) associated to each LOD, ranging between three and 51 mm of tolerance, considering it as the allowable dimensional deviation in the deliverable from truth (that has been obtained by some more precise other means). In the case of a point cloud, the guide specifies that the distance between two points from the model must be compared to the true distance between the same two points, and be less than or equal to the specified tolerance; the guide also defines the area of interest as a hierarchical system of scale in which each scan is registered, depending on the LOD. In Table 3, we summarize the data quality parameters defined by the GSA for registering point clouds.

**Figure 7:**
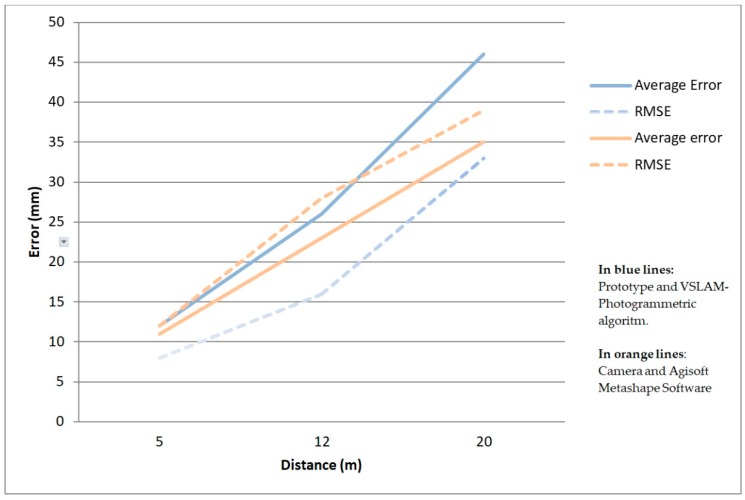
Graphic on the evolution of the average errors and RMSEs for the distances of 5, 12 and 20 m from the camera to the monument. The results are shown for both systems: Prototype and VSLAM-photogrammetric algorithm and Canon camera with Agisoft Metashape software.

**Figure 8:**
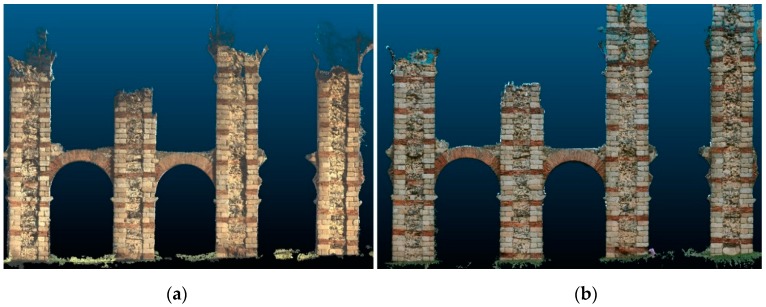
Comparison between points clouds resulting from both systems (with a data capture distance of 12 m): (**a**) Prototype and VSLAM-photogrammetric algorithm; and (**b**) Canon camera EOS 1300D with Agisoft Metashape software.

In order to complete the study, other photogrammetric system was analyzed under conditions similar to the prototype (Figure 7). The camera used was a Canon EOS 1300D and the lens was an EFS 18–55 mm, but we only used the focal length of 18mm for this experiment. Multiple images were taken in this experiment for each distance (35 images for 5 m, 41 images for 12 m and 43 images for 20 m) and the camera was configured with a resolution of 2592 pixels × 1728 pixels with the aim of comparing the results in an equitable way with the proposed approach which have a similar image resolution. The reflex camera´s parameters (shutter, diaphragm, ISO, etc.) were chosen in automatic mode during the test to match the conditions to the prototype test. The pictures were taken standing on the same trajectories previously followed by the prototype, at the same distances from the aqueduct: 5, 12 and 20 m. These circumstances increase the time consumed in the field during the data capture, as can be seen in the Table 4, because the user must focus each image and ensure that the picture has been taken with enough overlap and quality. On the other hand, the prototype cameras also have a configuration with automatic parameters which allowed the user, along with the methodology used, to make a continuous capture, without stopping to take the images. The images of the Canon camera were processed using the software Agisoft Metashape 1.5.4 [60] which is commercialized by the company Agisoft LLC, sited in St. Petersburg, Russia (Figure 8).

Point cloud density for each system was measured. Two points clouds, one for each system, were processed using the same 10 images of the aqueduct at a distance of 5 m. The density [37] of the point cloud was 328 points/dm^2^ for the proposed prototype system and 332 points/dm^2^ for the Agisoft Metashape photogrammetric software.

With the prototype and VSLAM-Photogrammetric algorithm we have computed the average error and the RMSE in each direction (*x*, *y*, and *z*) of each data capture distance, that are listed in Table 5, with overall accuracies of 12, 26 and 46 mm for 5, 12 and 20 m respectively and the RMSEs on each axis ranging between 5 to 8 mm (5 m), 10 to 21 mm (12 m) and 30 to 38 mm (20 m) (Figure 7) which satisfied the error tolerance of ‘level 1’ (51 mm) for data capture distances from 12–20 m and ‘level 2’ (13 mm) for data capture distances about 5 m.

**Figure 9:**
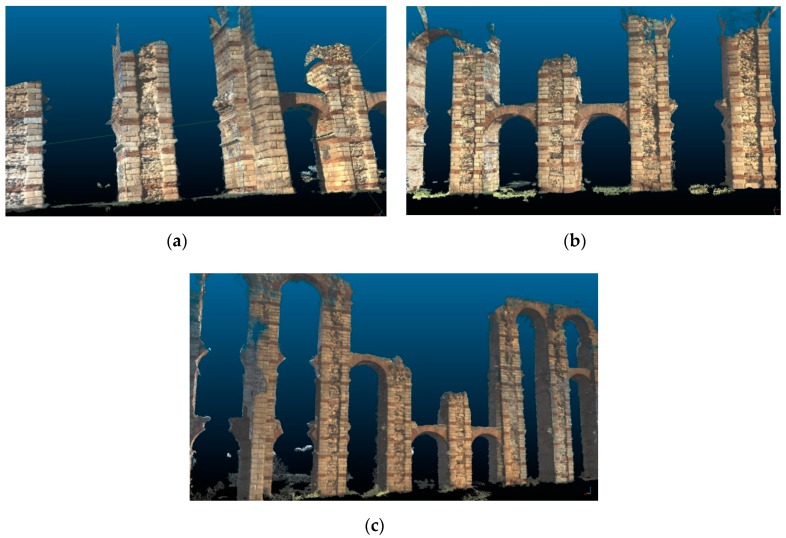
Points clouds resulted at the distances established in the experimental test: (**a**) 5 m; (**b**) 12 m; and (**c**) 20 m. The images show the central part of the color points clouds that resulted from the test. The points clouds have not been filtered or edited.

The point clouds obtained at the different distances of observation shown in Figure 9. Small holes or missing parts can be seen in those points clouds. This occurs due to the camera’s trajectory, since it needs to focus directly on all the desired areas and capture a minimum number of images to perform optimal triangulation. No filter has been applied in the results shown in Figure 9.

## 4. Conclusions

The major innovations of this study are as follows: First, the proposed approach for the 3D data capture and the implementation of the VSLAM-photogrammetric algorithm has been materialized in a functional and low-cost prototype, which has been checked in an experimental test, the results of which have been presented in the context of the BIM work environment.

Second, the results obtained in the experimental test comply with the precision requirements of the GSA BIM Guide for 3D Imaging for point cloud capture work with a resolution (minimum artifact size) of 152 mm × 152 mm, for observation distances of approximately 20 m. For distances between 5 and 12 m, we saw that better accuracies and resolution results were achieved.

Third, the possibility of using the instrument at different distances facilitates the data capture in shaded areas or areas with difficult access. This, together with the fact that the device has been designed for outdoor data collection, makes it suitable for urban design and historic documentation, which are usually carried out in outdoor environments, registering information for plans, sections, elevations and details and 3D point cloud in PLY format (positioning: *x*, *y*, *z* and color: R, G, B), following the GSA PBS(Public Building Service) CAD standards (2012) and the GSA BIM Guide for 3D Imaging Standards.

In order to increase the knowledge of the proposed approach, it has been compared with a well-known photogrammetric methodology consisting of a Reflex Canon 1300D camera and the software Agisoft Metashape. The results of the comparison test have provided interesting conclusions:

1. The accuracy results of both methods are similar as can be seen in Table 5. Although the average error is slightly higher in the proposed approach, the RMSE is a bit lower than with the Agisoft Metashape methodology. This indicates a small, but greater dispersion of the points of the proposed approach in respect to the Agisoft software. But as can be seen in the results, this factor does not imply an increase of RMSE, but this error is slightly less in the proposed approach in relation to Agisoft software.
2. The processing time was a bit higher in the proposed approach for the distances of 5 and 12 m but not for 20 m, for which the time was slightly less. The differences are not significant, in our opinion, and indicate that the proposed method optimizes the number of images extracted and the photogrammetric process, thus equating well-known procedures such as the use of a Reflex camera and the Agisoft Metashape software.
3. In our opinion, the greatest improvement occurred in the data capture field. The user does not worry about how to use the camera or where to take the picture, because in the proposed approach, the capture is continuous and the system chooses the images automatically, as is explained in Section 2. In this way, the learning curve changes significantly, provided the user doesn´t need to have previous knowledge about photography or photogrammetry. For this reason, the proposed approach here described, reduces significantly the time spent in the field, as can be seen in Table 4.

A new handheld mobile mapping system based on images have been presented in this paper. This proposed methodology does not adversely affect the known photogrammetric process (accuracy, processing time, point cloud density) but it proposes a new, easier and faster way to capture the data in the field, based on continuous data capture and fully automatic processing, without human intervention in any phase.

## Figures and Tables

**Table 1:** Main technical characteristics of cameras used in the prototype (from the Imaging Source Europe GmbH company).

Model	Resolution (Pixels)	Megapixels	Pixel Size (µm)	Frame Rate (fps)	Sensor	Sensor Size	A/D (bit)
DFK 42AUC03	1280 × 960	1.2	3.75	25	Aptina MT9M021 C	1/3”CMOS	8
DFK 33UX264	2448 × 2048	5	3.45	”8	Sony IMX264	2/3” CMOS	8/12

**Table 2:** Main technical data of lenses used in the prototype (from the Imaging Source Europe GmbH company and FUJIFILM Corporation).

Model	Focal Length (mm)	Iris Range	Angle of view (H × V)
TIS-TBL 2.1 C	2.1	2	97° × 81.2°
Fujinon HF6XA–5M	6	1.9–16	74.7° × 58.1°

**Table 3:** Data quality parameters defined by U.S. General Services Administration (GSA) for registering point clouds. (Unit: Millimeters).

Level of Detail (LOD)	Level of Accuracy (LOA, Tolerance)	Resolution	Areas of Interest (Coordinate Frame, c. f.)
Level 1	±51	152 × 152	Total Project area (Local or State c. f.)
Level 2	±13	25 × 25	e.g., building (local or project c. f.)
Level 3	±6	13 × 13	e.g., floor level (project or instrument c. f.)
Level 4	±3	13 × 13	e.g., room or artifact (instrument c. f.)

**Table 4:** Comparison between the proposed approach and the camera with Agisoft Metashape software in regards to the time spent in the field for data capture and processing time using the same laptop (Intel core i7 7700 HQ CPU processor, 16Gb RAM, Operative System Windows 10 Home). Distance values are measured from the camera to the monument.

System	Data Capture Distance (m)	Data Capture Time (min)	Processing Time (min)
**Prototype and Visual Slam (VSLAM)-Photogrammetric Algorithm**	5	4.25	80
	12	4.53	85
	20	4.65	99
**Canon Camera and Agisoft Metashape Software**	5	7.83	72
	12	9.08	80
	20	9.50	89

**Table 5:** This table compares the accuracy assessment results with the root mean square errors (RMSEs) and average errors for data capture distances from 5 to 20 m from the camera to the monument, between the prototype and VSLAM-photogrammetric algorithm and the Canon camera with Agisoft Metashape software. The RMSE error values have been computed in the three vector components: *X*, *Y* and *Z*.

	Methodology							
	Prototype and VSLAM-Photogrammetric Algorithm				Canon Camera and Agisoft Metashape Software			
**Distance 5 m**								
	**Error Vector *X* (mm)**	**Error Vector *Y* (mm)**	**Error Vector *Z* (mm)**	**Error (mm)**	**Error Vector *X* (mm)**	**Error Vector *Y* (mm)**	**Error Vector *Z* (mm)**	**Error (mm)**
**Average Error**				12				11
**RMSE**	5	8	8	8	4	9	8	12
**Distance 12 m**								
**AVERAGE Error**				26				23
**RMSE**	21	16	10	16	12	18	17	28
**Distance 20 m**								
**Average Error**				46				35
**RMSE**	32	30	38	33	18	24	24	39
